# Magnetic resonance quantification of regional blood flow and oxygen delivery to the brain, gut, kidneys, and lower extremities in adolescents with a Fontan circulation compared to biventricular controls

**DOI:** 10.1016/j.jocmr.2025.101907

**Published:** 2025-05-04

**Authors:** Jennifer Romanowicz, Sungho Park, Jenifer Bunn, Roni M. Jacobsen, Brian Fonseca, Jenny E. Zablah, Erin K. Englund, Alex J. Barker, Jesse A. Davidson

**Affiliations:** a Department of Pediatrics, Section of Cardiology, Children’s Hospital Colorado and University of Colorado Anschutz, 13123 E 16th Ave, Aurora, Colorado 80045, USA; b Department of Radiology, Section of Pediatric Radiology, Children's Hospital Colorado, University of Colorado Anschutz, 13123 E 16th Ave, Aurora, Colorado 80045, USA

**Keywords:** Congenital heart disease, Single ventricle, Total cavopulmonary anastomosis, MRI, Peak VO_2_, Exercise performance

## Abstract

**Background:**

Accumulation of progressive extracardiac disease is nearly universal for patients with single ventricle heart disease palliated to a Fontan circulation; however, etiologies are poorly understood. Limited flow reserve in the Fontan circulation may underlie extracardiac disease found in Fontan physiology through reduced oxygen and nutrient delivery to the tissues. This study aimed to determine regional flow volumes and oxygen delivery to key organ systems in children and adolescents with a Fontan circulation.

**Methods:**

In 17 Fontan subjects and 14 biventricular controls, regional arterial flow volumes to the carotid, celiac, superior mesenteric, renal, and iliac arteries were quantified with magnetic resonance imaging. Arterial oxygen content was calculated using subject hemoglobin level and pulse oximetry, and regional oxygen delivery was calculated using regional flow volume and oxygen content for the above-listed arteries. Cardiac output was measured from ascending aorta flow, systemic blood flow from the caval veins, and aorto-pulmonary collateral flow was calculated as the difference between the two. Flows were compared between groups (t-test) and associations were analyzed between flows and with maximal exercise performance on clinical cardiopulmonary exercise testing (Pearson correlation).

**Results:**

On average, renal and iliac arterial flows were lower in the Fontan group, compared to controls. Carotid, celiac, and superior mesenteric arterial flows were preserved in the Fontan group. Arterial oxygen content was equivalent between groups, and thus, regional oxygen delivery followed the same pattern as regional flows. Cardiac output was no different between groups, but systemic blood flow was lower in Fontans due to loss of flow to aorto-pulmonary collaterals. Systemic blood flow correlated with iliac flow such that those with the lowest systemic flow had the least amount of iliac flow. Celiac arterial flow correlated with percent-predicted peak oxygen consumption on exercise testing.

**Conclusion:**

Our results are consistent with a limited flow reserve in the Fontan circulation with sacrifice of iliac arterial flow as global systemic blood flow decreases. Importantly, these data were measured with subjects supine and at rest. Future work requires the addition of exercise to determine how flow to specific organs is affected by increasing metabolic demand from the extremities.

## Graphical abstract

**Figure:**
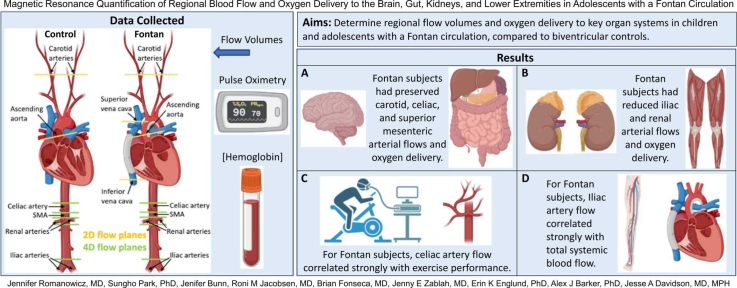

## 1 Introduction

Children with single ventricle heart disease undergo palliation to the Fontan circulation, which eliminates the sub-pulmonary ventricle, creating passive pulmonary blood flow and chronic systemic venous hypertension. Accumulation of extracardiac disease is nearly universal after the Fontan procedure, contributing to reduced quality and length of life [1]. The pathophysiology of Fontan-associated extracardiac disease is poorly understood.

The Fontan circulation is a low cardiac output (CO) state [1], [2]. Limited flow reserve has been demonstrated, especially with exercise or significant collateral vessels [3], [4], leading to insufficient oxygen/nutrient supply to the tissues. Inadequate tissue perfusion and venous congestion are hypothesized to underlie many Fontan-associated extracardiac pathologies [2]. However, the characterization of regional blood flow and oxygen delivery in the Fontan circulation is limited.

Our study aimed to utilize magnetic resonance imaging (MRI) to quantify regional flow volumes and oxygen delivery to the brain, gut, kidneys, and legs in adolescents with a Fontan circulation, and to compare these findings to biventricular controls.

## 2 Methods

This study was approved by the Colorado Multiple Institutional Review Board. Informed consent was obtained.

Study participants were identified from the cardiovascular magnetic resonance (CMR) imaging schedule. For the Fontan cohort, all single ventricle lesions were included. For the control group, inclusion criteria were normal cardiac anatomy, normal biventricular systolic function, and at most mild valvular disease. Exclusion criteria for both groups were anesthesia, anxiolysis, or supplemental oxygen during the CMR.

### 2.1 Clinical data

Clinical data were obtained from the electronic health record and included demographics, exercise testing results, and cardiac anatomy. Exercise data were taken from the test closest to the CMR. Before the CMR, participants were asked about the timing of their last food intake, and blood was drawn for hemoglobin measurement. Arterial oxygen saturation was measured via pulse oximetry during the CMR.

### 2.2 Global flow

**Fig. 1:**
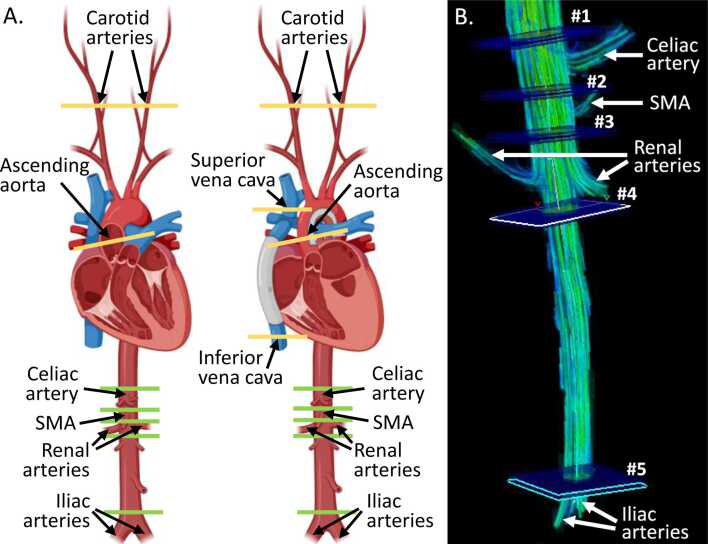
Location of flow measurements for control (left) and Fontan (right) subjects (A) and representative abdominal 4D flow image (B). 2D phase-contrast (PC) sequences were planned in planes shown in yellow to measure carotid arterial and ascending aorta flow in both groups and superior and inferior venae cavae in the Fontan group. Abdominal flow volumes were obtained from a single 4D PC sequence covering the descending aorta from the diaphragm to the aortic bifurcation. Planes where through-plane flow volume was measured on post-processing are shown in green (A) and labeled #1–5 (B). Descending aorta flow volumes above/below the branches of interest were subtracted to determine flow to the celiac, superior mesenteric (SMA), and renal arteries. Iliac arterial flow was measured directly just above the aortic bifurcation. Panel A was created in https://BioRender.com. Panel B demonstrates flow as velocity-coded streamlines (created with EnSight, CEI, Kansas City, Missouri). *2D* two-dimensional, *4D* four-dimensional

Flow volumes were quantified on a 1.5T scanner (Philips Ingenia, Best, The Netherlands) after the injection of gadolinium-based contrast (Gadovist 1.0, Bayer, Mississauga, Ontario, Canada) (Fig. 1). Post-processing was completed in Circle CVI (Circle Cardiovascular Imaging, Calgary, Alberta, Canada). CO was measured by ascending aorta flow. Systemic blood flow (Qs) was calculated by adding inferior and superior venae cavae flows. Aortopulmonary collateral (APC) flow was calculated by subtracting Qs from CO [3]. Flows were obtained with two-dimensional (2D) phase-contrast (PC)-MRI and indexed to body surface area (BSA), which was calculated using the Haycock formula.

### 2.3 Regional flow

Carotid arterial flow was quantified by 2D PC-MRI. The remaining flows were extracted from a 4D flow PC-MRI sequence covering the descending aorta from the diaphragm to the aortic bifurcation (voxel size 2.5 mm^3^, echo time 2.8 ms, repetition time 4.8 ms, flip angle 14°, velocity sensitivity encoding 120 m/s, 18 reconstructed cardiac phases, nominal temporal resolution 48 ms). Celiac, superior mesenteric, and renal arterial flow volumes were calculated by subtracting aortic flow volumes above and below the branch(es) of interest. Iliac arterial flow volume was measured just above the aortic bifurcation. Eddy current correction and noise filtering were performed during post-processing.

### 2.4 Regional oxygen delivery

Oxygen delivery is the product of arterial flow volume and blood oxygen content. To calculate regional oxygen delivery, we utilized our regional arterial flow volumes. Blood oxygen content was calculated as follows: oxygen content (mL oxygen/L blood) = 1.34 mL oxygen/gm hemoglobin * hemoglobin concentration (gm/L) * oxygen saturation/100. Because subjects were in room air, dissolved oxygen was presumed negligible and omitted from the oxygen content calculation.

### 2.5 Statistical analysis

Normal distributions of the data were confirmed by Shapiro-Wilk normality tests. Differences in flow and oxygen delivery between groups were assessed by unpaired Student’s t-tests with a significance threshold of p ≤ 0.05. Relationships between flows or with exercise performance were assessed by Pearson correlations. Multiple comparisons adjustments were not performed as the intention of this initial pilot study is to be signal inclusive as a hypothesis-generating step for future investigations.

## 3 Results

Seventeen Fontan subjects and 14 controls were enrolled between August 2023 and October 2024. There was no difference in sex, age, weight, or BSA between groups; however, controls were taller (Table 1). The Fontan group had lower oxygen saturation and higher hemoglobin concentration such that there was no difference between groups in arterial oxygen content. Median time between exercise testing and CMR was 12.5 days (interquartile range 2.5–115.5 days, total range 0 days to 7 months).

**Table 1:** Demographics and baseline characteristics.

	Control (N = 14)	Fontan (N = 17)	p-value
Male sex	6 (43%)	9 (53%)	0.58
Age (years)	16.2 (0.8)	14.2 (0.9)	0.10
Weight (kg)	65.6 (8.8)	62.2 (5.4)	0.74
Height (cm)	170.6 (3.7)	157.5 (3.5)	**0.016**
Body surface area (m^2^)	1.74 (0.13)	1.64 (0.09)	0.49
Oxygen saturation (%)	96.2 (0.3)	89.4 (0.8)	**<0.0001**
Hemoglobin (gm/dL)	14.7 (0.4)	16.0 (0.3)	**0.016**
Oxygen content (mL O_2_/L)	189.7 (4.5)	192.0 (4.4)	0.73
Cardiac morphology			
Single right ventricle		7 (41%)	
Single left ventricle		10 (59%)	
Indication for CMR			
Bicuspid aortic valve	7 (50%)		
Other aortopathy risk	2 (14%)		
Family history cardiomyopathy	3 (21%)		
Other	2 (14%)		

Data are presented as N (%) or mean (standard error of the mean).

*CMR* cardiovascular magnetic resonance

Significant p-values are shown in bold font.

### 3.1 Global flow

**Fig. 2:**
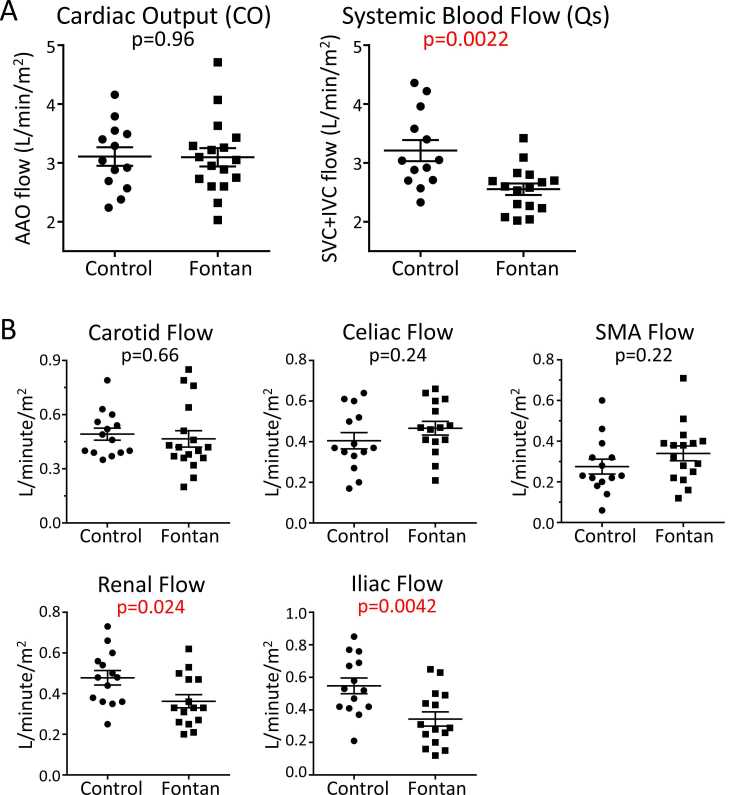
Global (A) and regional (B) flow volume comparisons between cases and controls. Data are displayed as mean with standard error of the mean. Unpaired t-tests were used to compare flows between groups and the p-value is displayed. All flows were indexed to body surface area. *SMA* superior mesenteric artery, *AAO* ascending aorta, *SVC* superior vena cava, *IVC* inferior vena cava

Qs was lower in the Fontan group compared to controls (Fig. 2A). There was no difference in CO between groups. Neither CO nor Qs was associated with exercise performance as measured by percent-predicted peak oxygen consumption (VO_2_).

### 3.2 Regional flow

**Fig. 3:**
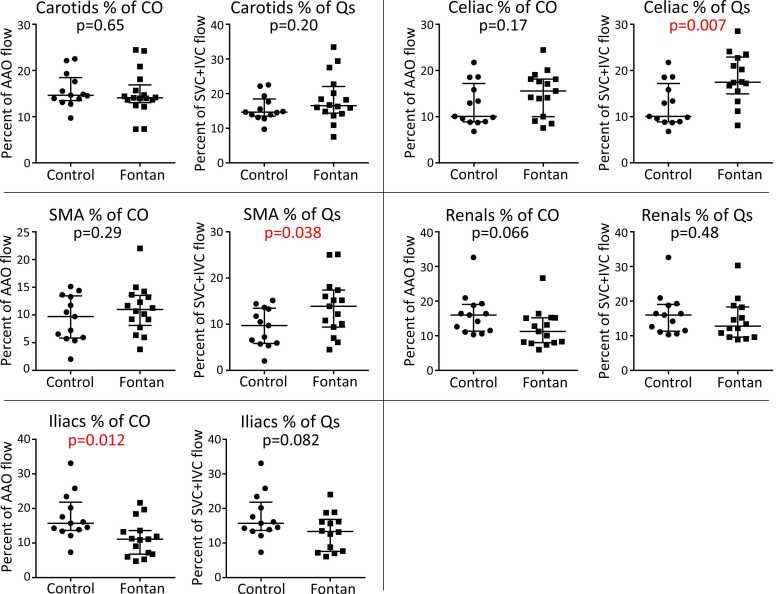
Regional flow volumes standardized to global flow. Data are displayed as mean with standard error of the mean. Unpaired t-tests were used to compare flows between groups and the p-value is displayed. Flows were standardized to either cardiac output (CO) which was calculated by ascending aorta (AAO) flow or systemic blood flow (Qs) which was calculated by adding superior (SVC) and inferior (IVC) venae cavae. *SMA* superior mesenteric artery

Renal and iliac flow volumes were lower in the Fontan group than in controls (Fig. 2B). There was no difference between groups for carotid, celiac, and superior mesenteric arterial flow. Celiac and superior mesenteric arterial flow accounted for a higher percentage of Qs for the Fontan group than controls (Fig. 3). Iliac arterial flow accounted for a lower percentage of CO for the Fontan group than controls. For both cases and controls, there was no relationship between iliac flow volume and height.

To assess for relationships between APC flow and regional flows in the Fontan group, regional flow volumes were standardized to CO and APC flow indexed to BSA, as previously described [3]. There were no significant associations between APC flow and any of the regional flows (Table 2).

**Table 2:** Association of aortopulmonary collateral (APC) flow with regional flows.

	r	p-value
Carotid flow (% of cardiac output)	0.02	0.94
Celiac flow (% of cardiac output)	−0.30	0.28
SMA flow (% of cardiac output)	0.10	0.71
Renal flow (% of cardiac output)	−0.29	0.30
Iliac flow (% of cardiac output)	−0.29	0.29

Pearson correlation r and p-values are presented for the relationship between aortopulmonary collateral flow and the listed variables. Regional flows are indexed to total cardiac output. APC flow is indexed to body surface area

*SMA* superior mesenteric artery

**Fig. 4:**
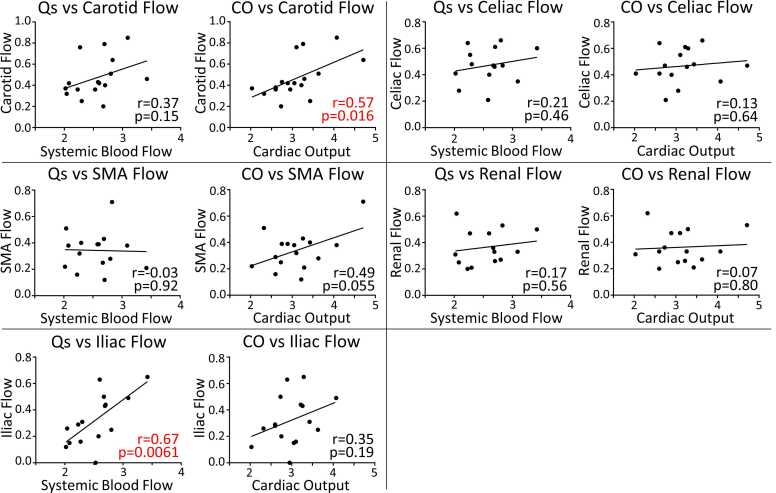
Associations between regional and global flows in the Fontan group. All flows are indexed to body surface area and measured in liters per minute per meter squared. The r and p values from the Pearson correlation are shown on the graph. A linear regression line is shown for each association. *CO* cardiac output, *Qs* systemic blood flow, *SMA* superior mesenteric artery

We evaluated for associations between global flow and regional flow (Fig. 4). In the Fontan group, carotid flow correlated strongly with CO, iliac flow correlated strongly with Qs, and there were no other significant correlations. For controls, none of the regional flows were associated with global flow.

**Fig. 5:**
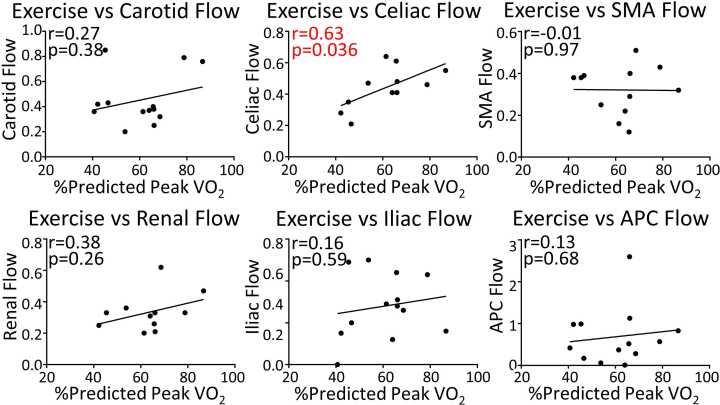
Associations between regional flows and exercise performance in the Fontan group. All flows are indexed to body surface area. The r and p values from the Pearson correlation are shown on the graph. A linear regression line is shown for each association. Exercise performance is represented by percent-predicted peak oxygen consumption (VO_2_). *SMA* superior mesenteric artery, *APC* aortopulmonary collateral

Finally, in the Fontan group, we assessed for relationships between regional flows and exercise performance as measured by percent-predicted peak VO_2_ (Fig. 5). Celiac flow correlated with exercise performance. There were no other significant associations.

### 3.3 Regional oxygen delivery

Regional oxygen delivery was lower to the renal and iliac arteries in the Fontan group compared to controls (Table 3). There was no difference between groups for the carotid, celiac, and superior mesenteric arteries.

**Table 3:** Regional oxygen delivery.

	Fontan	Control	p-value
Carotid arteries	88.9 (8.3)	93.0 (6.0)	0.70
Celiac artery	89.7 (7.3)	76.0 (7.4)	0.19
Superior mesenteric artery	65.2 (6.5)	52.5 (7.2)	0.20
Renal arteries	70.0 (6.7)	91.5 (7.8)	**0.046**
Iliac arteries	62.8 (8.7)	104.7 (10.2)	**0.0041**

Data are presented as mean (standard error of the mean). All values are presented in milliliters of oxygen per minute per meter squared. Significant p-values are shown in bold font.

## 4 Discussion

Extracardiac disease is nearly ubiquitous in the Fontan population; however, its etiology is poorly understood. We sought to quantify regional flow volumes and oxygen delivery to elucidate the metabolic substrate seen by end-organ systems in the Fontan circulation as a possible nidus for pathophysiology. We found no difference in CO between groups; however, loss of flow to collaterals resulted in lower Qs in the Fontan group. Despite lower Qs, regional flow volumes to the carotid, celiac, and superior mesenteric arteries were preserved. Renal and iliac flow volumes were decreased for the Fontan group, which may be explained by increased renal and iliac impedance demonstrated by Hauser et al., in an adult Fontan population [5]. Because oxygen content was equivalent between groups, regional oxygen delivery mirrored regional flows.

Reduced CO is described as a hallmark of the Fontan circulation [1]. Presumably, the intention of this description lies in the Fontan circulation’s reduced ability to augment CO with activity. However, it is worth explicitly stating that in our adolescent Fontan cohort, CO matched that of biventricular controls while at rest, which is a departure from the traditional description of Fontan physiology, but consistent with other recent studies [6]. Previous studies of abdominal regional flow in the Fontan circulation have been mixed with some demonstrating reduced celiac and superior mesenteric flow for Fontan subjects [7], [8] and another demonstrating no difference from controls [5]. In contrast, our results demonstrate a trend toward increased flow in these arteries. Further research is necessary to determine the source of heterogeneity in splanchnic blood flow in the Fontan circulation.

Our results are consistent with a limited flow reserve in the Fontan circulation. Iliac flow correlated with overall Qs in the Fontan group, a relationship not observed in controls. Therefore, in Fontan participants, those with the lowest Qs had the least iliac flow. The iliac arteries primarily supply the lower extremity muscles, and thus our data suggest that skeletal muscle perfusion is reduced to maintain perfusion to crucial organ systems. Importantly, our subjects were imaged at rest. Next steps include measuring regional flow volumes with the addition of exercise to stress the perfusion reserve observed in our Fontan cohort.

The relationship between increased celiac flow and better exercise performance is not entirely clear. In normal exercise physiology, splanchnic flow decreases proportionally to exercise intensity to provide flow reserve to active limbs [9]. It is possible that those Fontan patients with greater celiac flow at rest are able to supplement flow reserve more robustly during exercise. This observation further underscores the need for regional flow analysis with exercise in the Fontan population.

### 4.1 Limitations

Limitations include the inability to control for different medications and complications within the Fontan group due to sample size. Heterogeneity observed within the Fontan group requires future exploration with a larger cohort to power within-group comparisons. Due to the sample size, this study may be vulnerable to both type 1 and type 2 errors and we encourage further studies to validate and expand upon our findings. Additionally, prandial status was collected, but controlling this variable was not part of our experimental design. Celiac and superior mesenteric artery flow volumes increase after a meal [10]; however, both peak in the first hour and none of our subjects had eaten in the 3 hours before the CMR. Thus, we expect this source of heterogeneity to be minimal. There was a statistically non-significant trend toward a younger Fontan cohort, and with unmeasured potential differences in pubertal status, it is possible there were differences in muscle bulk or other hemodynamic contributors that were not completely controlled for by standardizing flows to BSA. The average oxygen saturation for the Fontan group was 89% which is at the lower end of the typical range for Fontan patients (90–95%); this may represent a negligible difference, inclusion of a younger cohort more likely to have a patent fenestration, the effects of moderate altitude (∼5400 ft), or an increased incidence of systemic-to-pulmonary venovenous collaterals or pulmonary arteriovenous malformations; any of which may affect generalizability. Finally, potential abdominal vascular variant anatomy may have gone undetected. Although the celiac trunk gives rise to its three main branches (gastric, hepatic, and splenic arteries) 89–94% of the time, one or both inferior phrenic artery arises from the celiac trunk in up to 40% of cases [11]. Our imaging resolution was not sufficient to assess the inferior phrenic artery origins. Additionally, supernumerary renal arteries occur in up to 30% of individuals [12]. Renal blood flow was measured as the difference in descending aorta flow above and below renal arteries, so the majority of supernumerary renal arteries would have been included as they arise near to each other. Rare small polar renal arteries that arise distant from the other renal arteries likely went undetected and were excluded from calculations.

### 4.2 Conclusions

The Fontan circulation is a state of limited perfusion reserve. At rest, Fontan patients experience reduced flow and oxygen delivery to the kidneys and lower extremities but maintain normal flow and oxygen delivery to the gut and brain. Future research evaluating regional flow and oxygen delivery with the addition of exercise is necessary to understand the full impact of this limited flow reserve on individual organ systems that are so vulnerable to disease in the Fontan population.

## Author contributions

**Jenny E. Zablah:** Writing – review & editing. **Brian Fonseca:** Writing – review & editing. **Alex J. Barker:** Writing – review & editing, Resources, Methodology, Formal analysis, Data curation, Conceptualization. **Erin K. Englund:** Writing – review & editing, Methodology. **Jesse A. Davidson:** Writing – review & editing, Writing – original draft, Visualization, Methodology, Funding acquisition, Formal analysis, Conceptualization. **Sungho Park:** Writing – review & editing, Data curation. **Jennifer Romanowicz:** Writing – review & editing, Writing – original draft, Visualization, Resources, Project administration, Methodology, Investigation, Funding acquisition, Formal analysis, Data curation, Conceptualization. **Roni M. Jacobsen:** Writing – review & editing. **Jenifer Bunn:** Writing – review & editing, Project administration, Data curation.

## Ethics approval and consent

Permission to conduct the study was granted by the Colorado Multiple Institutional Review Board on April 9, 2023 (COMIRB 23-0451). Written informed consent was obtained from each subject (or subject's legal guardian with subject assent if younger than 18 years old) at enrollment for both cases and controls. The research was conducted in accordance with the principles embodied in the Declaration of Helsinki and in accordance with local statutory requirements.

## Declaration of competing interests

The authors declare that they have no known competing financial interests or personal relationships that could have appeared to influence the work reported in this paper.

## Data Availability

The data that support the findings of this study are available from the corresponding author, J.R., upon reasonable request.
